# Implementation of podiatry telephone appointments for people with rheumatic and musculoskeletal diseases

**DOI:** 10.1186/s13047-020-00441-9

**Published:** 2021-01-07

**Authors:** J. L. Palmer, H. J. Siddle, A. C. Redmond, B. Alcacer-Pitarch

**Affiliations:** 1 College of Health, Wellbeing and Life Sciences, Sheffield Hallam University, Sheffield, UK; 2 First Contact Physiotherapy Practitioner, Primary Care Sheffield, Darnall Primary Care Centre, Sheffield, UK; 3 Leeds Institute of Rheumatic and Musculoskeletal Medicine, University of Leeds, 2nd Floor, Chapel Allerton Hospital, Leeds, LS7 4SA UK; 4 Podiatry Department, Leeds Teaching Hospitals NHS Trust, Leeds, UK; 5 NIHR Leeds Biomedical Research Centre, Leeds Teaching Hospitals NHS Trust, Leeds, UK

**Keywords:** Audit, Rheumatology, Telephone, Follow-up, Clinic, Appointment, Foot, COVID-19

## Abstract

**Background:**

Foot health problems are common in the general population, and particularly so in people with rheumatic and musculoskeletal disorders (RMD). Several clinical guidelines state that people with RMDs should have access to foot health services, although service capacity is often limited. The current COVID-19 pandemic has increased the need for alternative ways to provide patient care. The aim of this clinical audit was to review a newly implemented telephone follow-up appointment service conducted within the Rheumatology Podiatry Department in Leeds, UK.

**Methods:**

Fifty-eight patients attending the Rheumatology Podiatry Department at Leeds Teaching Hospitals NHS Trust were contacted by telephone approximately 6–8 weeks following initial intervention. During the telephone consultation, all patients were asked pre-defined questions relating to their symptoms, intervention efficacy, the need for further appointments and their preference for the type of consultation. To assess the cost of the telephone consultation the number of attempts needed in order to make successful contact, the duration of the call and the number of telephone follow-up appointments completed in a working day were also recorded.

**Results:**

Twenty-five patients (43%) were successfully contacted within the 6–8 weeks stipulated time frame and were included in the analysis. Of the 25 contacted, twelve (48%) patients were successfully contacted on the first attempt. Ten (40%) were successfully contacted on the second attempt. The remaining three patients (12%) required 3 or more attempts to make successful contact. Telephone consultations were estimated not to last longer than 10 min, including notes screening and documentation. Eleven patients (44%) reported an improvement in their symptoms, thirteen (52%) reported no change and one patient (4%) reported their symptoms to be worse.

**Conclusion:**

Telephone follow-up consultations may be a potentially cost-effective alternative to face-to-face appointments when implemented in a Rheumatology Podiatry Department, and provide an alternative way of providing care, especially when capacity for face-to-face contact is limited. The potential cost saving and efficiency benefits of this service are likely to be enhanced when telephone consultations are pre-arranged with patients.

**Supplementary Information:**

The online version contains supplementary material available at 10.1186/s13047-020-00441-9.

## Background

Foot health complaints are common worldwide [1]. Garrow et al. (2004) reported that in the United Kingdom (UK) in particular, 63% of people experience a foot health complaint at some point in their lives and 10% report ongoing, disabling foot pain [1]. People with rheumatic and musculoskeletal disorders (RMD) are amongst those who are most affected by foot problems. Over 90% of people with rheumatoid arthritis (RA) and over 80% of people with systemic lupus erythematosus experience foot health problems [2–4]. High rates of disabling foot pain are also seen in people with osteoarthritis (9.6%) and systemic sclerosis (67%) [5, 6].

In the UK there are generic and disease specific national and local guidelines recommending that people with RMDs and foot problems should have access to foot health services, including the Arthritis and Musculoskeletal Alliance (ARMA) and the UK Podiatry Rheumatic Care Association (PRCA) Standards of Care for People with Musculoskeletal Health Problems [7, 8]. The National Institute for Clinical Excellence (NICE) guidelines state that everyone with a diagnosis of RA who reports foot health problems should have access to podiatry services for both assessment and treatment [7–9]. NICE also advocate specific treatments such as provision of foot orthoses for the treatment of foot problems in people with RA [9]. Foot orthoses are also used in other rheumatic diseases to reduce foot pain and improve function [4, 10, 11]. In addition to recommendations made relating to specific treatment interventions, local guidelines, such as those developed by the North West Clinical Effectiveness Group (NWCEG), suggest telephone follow-up consultations should be offered to patients as part of their ‘Gold Standard requirements for a Podiatry Service’ [12]. The recommended telephone service aims to provide patients with timely access to advice related to their care as well as part of an ongoing assessment process, while minimising hospital visits. These local guidelines are also in-keeping with NICE guidance which states that people with RA should have the opportunity for review appointments that suit their individual needs [9].

Advocating the use of telephone and online appointments within various clinical guidelines is reassuring to clinicians during recent times in the wake of COVID-19. Since the declaration of a worldwide pandemic by the World Health Organisation on March 11th, 2020, there has been an unprecedented shift towards alternative methods of consultation within the health care sector. These measures have been implemented in order to minimise the potential for transmission of the virus through non-essential hospital attendance and minimising face-to-face contact between patients and clinicians [13]. Prior to the COVID-19 outbreak, the recommendations made by ARMA, NICE and the NWCEG had been implemented in the Rheumatology Podiatry Department at Leeds Teaching Hospitals NHS Trust (LTHT) UK. In LTHT, patients presenting with RMDs and foot problems have access to foot health services for assessment and treatment of their foot complaints. After treatment, patients require a follow-up consultation for therapy-review. In the LTHT Podiatry department, these reviews have historically all been undertaken during a face-to-face clinical appointment. However, in view of the NWCEG local recommendations, the podiatry team started to implement a telephone follow-up consultation for patients in whom the therapy provided does not necessarily require a face-to-face follow-up appointment and for patients who live outside of the region or who had difficulty in attending during the typical working day. This approach to patient follow-up offers opportunities to minimise the burden of unnecessary time and travel to patients and to improve capacity within the service. It also offers the additional benefit of minimising the need for face-to-face consultations during the current pandemic where in-person contact has been restricted to essential care to those as risk of life or limb only.

The aim of this audit was to review a newly implemented telephone follow-up appointment service conducted within the Rheumatology Podiatry Department in Leeds, UK.

## Methods

### Patients

Patients attending the Rheumatology Podiatry Department at LTHT, Leeds, from January 2015 to May 2015 were included in the audit. Fifty-eight were identified as potential patients. Four of these were identified prospectively while attending the Rheumatology Podiatry Department and 54 retrospectively through screening of the previous eight-week clinic lists. Patients were over the age of 18 at the time of assessment, were under the care of a rheumatologist, and received podiatry interventions such as foot orthoses. Those patients unwilling to participate or unable to understand English were excluded as it was not viable to provide patients with a translator for telephone review at home.

### Audit tool

The audit was registered with and authorised by the LTHT Quality Improvement Team. As this was not a primary research project, ethical approval was not required.

The audit tool consisted of a total of four questions (see Additional file 1: Appendix 1) capturing the magnitude of the patients’ perceived change in symptoms post treatment using a Global Rating of Change (GRC) Score. This was in the form of a 15-point modified Likert scale ranging from − 7 (‘A very great deal worse’) to + 7 (‘A very great deal better’). This can be found in Additional file 1: Appendix 1 [14–16]. The telephone questionnaire was developed by the team, consisting of a specialist musculoskeletal physiotherapist (JLP), two experienced rheumatology podiatrists (BAP and HJS) and an academic podiatrist (ACR); each with significant research experience in the relevant subject.

### Therapy-review follow-up consultation

Patients received a follow-up telephone consultation six to eight weeks after receiving podiatry intervention. The follow-up period was set at between six to eight weeks to allow adequate time for the therapy provided to offer potential clinical benefit. During the consultation, patients were asked questions 1, 3 and 4 of the audit questionnaire (Additional file 1: Appendix 1). Only those patients reporting a change in symptoms (i.e. better or worse), were asked question 2 of the telephone consultation questions (Additional file 1: Appendix 1). Patients reporting their symptoms to be better or worse were asked to rate their change on one of the 7 corresponding points of the 15-point modified-Likert Scale (i.e. -1 to − 7 for those reporting their symptoms to be worse and + 1 to + 7 for those reporting their symptoms to be better). Patients who reported their symptoms to be the same were recorded as a zero (‘No change’) for their GRC Score.

In order to assess the costs of the telephone follow-up consultation, the number of attempts made prior to making successful contact with patients, an estimate of the duration of the call and the number of telephone follow-up appointments completed in a working day were recorded. This allowed financial implications to be calculated based on the additional number of patients seen in a working day.

## Results

**Fig. 1:**
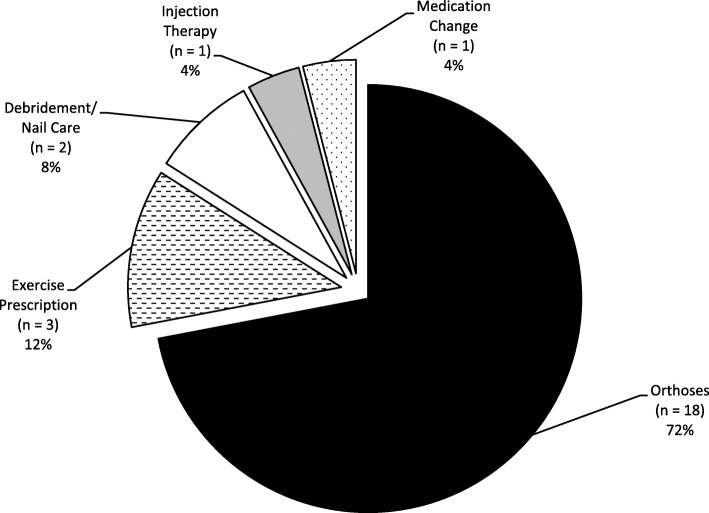
Intervention Delivered

Fifty-eight consecutive patients attending Rheumatology Podiatry Department at LTHT with RMDs were identified as suitable for inclusion in the audit. Thirty-three were excluded because either they had received a face-to-face follow-up appointment or the time post-intervention exceeded eight weeks. Thus, 25 patients were appropriate for telephone review (23 female and 2 male); mean age 55.5 years (range 25–82). The majority of patients (18/25; 72%) received foot orthoses as the primary intervention for their foot health problem (Fig. 1). Three patients (12%) were managed with exercise prescription, two (8%) received debridement/ nail care, one (4%) patient was treated with a steroid injection and one (4%) patient had a medication change (Fig. 1).

### Telephone contact

**Fig. 2:**
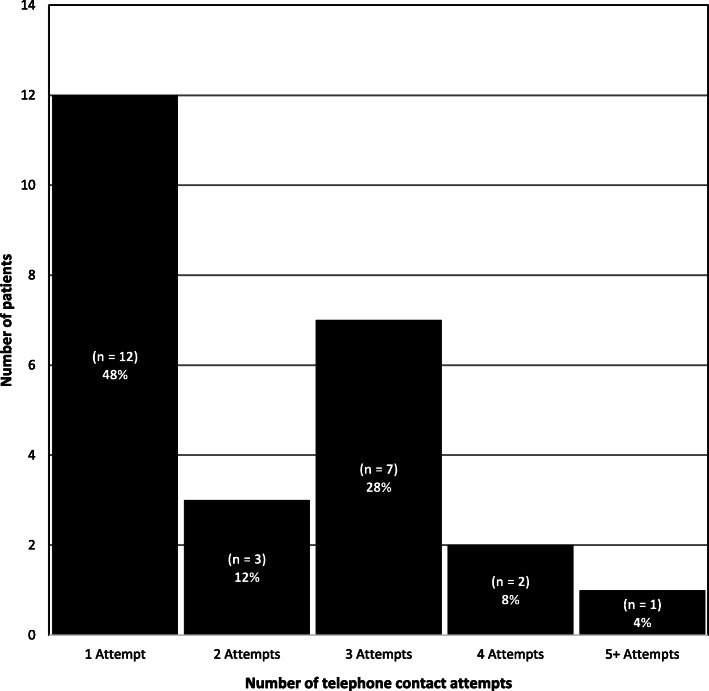
Telephone contact attempts

Of the 25 patients included in the data analysis, 12 (48%) were contacted successfully on the first attempt; ten (40%) patients required a second or third telephone call before contact was successful; 3 (12%) on the second and 7 (28%) on the third attempt. Two patients (8%) were successfully contacted on the fourth attempt and the remaining 1 (4%) patient, required five attempts (Fig. 2). Only questions presented in Additional file 1: Appendix 1 were asked during telephone contacts. Although formal data was not collated as to the duration for each contact, no call lasted longer than 10 min, this included screening medical notes and documenting patient responses.

As the majority of patients were contacted ad hoc following the retrospective screening of clinic lists, some already had face-to-face follow-up appointments pre-booked, either with a podiatrist or rheumatologist. This made the final two questions relating to the booking of further appointments (see Additional file 1: Appendix 1) redundant, thus these were not included in the analysis. Although this audit did not attempt to formally detail missing data or recruitment success there were no obvious systematic patterns.

### Financial costs associated with follow-up telephone consultation

At the time of conducting this audit, costs according to clinical tariffs at LTHT were £35.80 per face-to-face follow-up appointment in the Rheumatology Podiatry Department. The costing attached to a telephone consultation was £24.00; a saving of £11.80 in comparison to that of a face-to-face follow-up appointment.

### Treatment outcomes

**Fig. 3:**
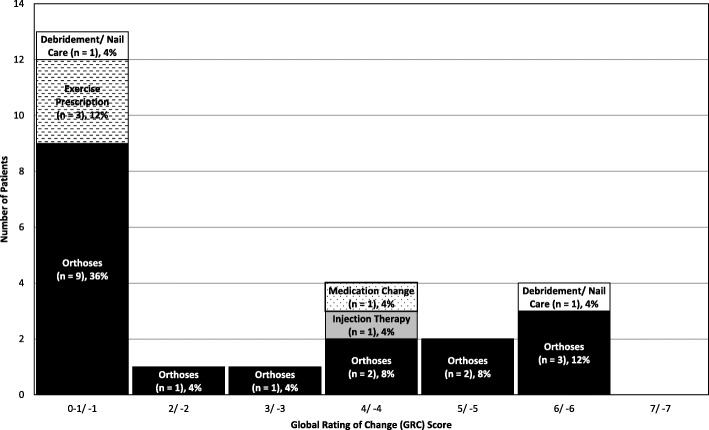
Global Rating of Change (GRC) Scores for specific interventions

During the follow-up telephone consultations 11 (44%) out of 25 patients reported an improvement in their symptoms (GRC of ≥2); eight (32%) receiving foot orthoses, one (4%) receiving debridement/ nail care, one (4%) receiving injection therapy and one (4%) undergoing a medication change. Thirteen (52%) patients reported their symptoms had not changed (GRC Score of − 1, 0 or + 1); nine (36%) received foot orthoses, three (12%) were managed with exercise prescription and one (4%) received nail care/ debridement. One patient (4%) reported worsening of their symptoms (GRC Score ≤ − 2) after being provided with foot orthoses (Fig. 3).

## Discussion

The aim of this clinical audit was to review a newly implemented telephone follow-up consultation service in the LTHT Rheumatology Podiatry Department. Due to the retrospective identification of most patients included in this audit, results relating to the need for further face-to-face consultations were not reported. However, the questions posed in the audit can be used to establish the need for further face-to-face consultations following clinical intervention, with the potential to have an impact on health care services and patients. This is pertinent given the current challenges faced in clinical practice surrounding the transition to virtual/ telephone consultations in the wake of the COVID-19 pandemic [13]. The success and efficiency of this service does however appear to be influenced by how these appointments are arranged with patients.

With regards to time efficiency, almost half of patients were successfully contacted on the first attempt with a further 40% being successfully contacted on either the second or third attempt. Only 12% required 4 or more contact attempts before being reached. It must however be taken into consideration that of the 25 patients identified as suitable for inclusion, only 4 (16%) were identified prospectively and would have had their telephone review pre-arranged; increasing the likelihood of making successful contact via telephone. All patients were also contacted during what is considered to be normal working hours. These factors could potentially explain why in our community dwelling sample 13 (52%) patients required more than 1 attempt to make successful contact. Taking this into consideration, it could be suggested that in order to make the system run efficiently and effectively, telephone follow-up calls should be pre-arranged in a similar manner to face-to-face appointments. In doing so, this will likely reduce the number of call attempts required before making successful contact.

Dudas et al. (2001) restricted the number of attempts to contact patients to three in their study investigating patient satisfaction and outcomes associated with telephone follow-up on discharge from hospital, with those exceeding this number being considered as lost to follow-up [17]. Of the 110 patients included in Dudas’ telephone follow-up group, 79 (72%) were contacted successfully within three attempts. During the current clinical audit, 22 of the 25 (86%) patients included were successfully contacted within the first three attempts. It must however be acknowledged that the small numbers in this clinical audit does reduce the external validity of findings and therefore limit any definitive conclusions [18]. Utilising a similar approach in the clinical setting to restrict the number of contact attempts to three for pre-arranged telephone appointments would serve to further reduce the time invested by the clinician and by virtue, improve efficiency. A further limitation of the approach adopted within the clinical audit was the identification of patients retrospectively through the screening of clinic lists. As a result, patients were not provided with an approximate date/ time for their telephone review. However, implementing this approach in the clinical setting would serve to mitigate this by providing patients with a designated telephone follow-up appointment after their initial face-to-face consultation.

Although data were not formally collected as to the exact duration of each telephone consultation as part of this clinical audit, contacts lasted less than 10 min per patient. This included time needed to obtain and screen medical notes and document the patient’s response to the audit questions (Additional file 1: Appendix 1). Based on a normal LTHT rheumatology podiatry clinic consisting of eight 20-min face-to-face follow-up appointments, a change to offering patients the opportunity to be reviewed by telephone may significantly improve clinic accessibility costs and reduce unnecessary patient visits to clinic. It must be acknowledged that for the purposes of this audit there was no deviation from the questions presented in Additional file 1: Appendix 1 and more routine patient-clinician interactions may require varying lengths of contact time in comparison to this.

Cost analysis at the time of initial data collection showed a potential saving of £11.80 per patient contact using telephone follow-up appointments. However, since the introduction of the Aligned Incentive Contract by the NHS Leeds Clinical Commissioning Group in April 2018, the costs associated with all patient contacts within the Rheumatology Podiatry Department at LTHT have been standardised across both telephone and face-to-face consultations. This potential cost saving benefit could be a factor for consideration for other trusts and departments where traditional tariff-based systems for patient contacts are still in operation.

Other potential financial savings for the NHS (not calculated in this audit), might be those related to reduced non-attendance rates, by patients who do not feel further appointments are necessary and would prefer a short telephone follow-up consultation instead of a face-to-face hospital appointment. There are also broader implications such as reducing the demand on NHS patient transport services often utilised by patients with poor mobility/ additional needs and who require transport to attend face-to-face consultations. The rheumatology podiatry service offered at Chapel Allerton Hospital (LTHT) provides care to patients across the region, with patients often travelling long distances to attend appointments. Following up such patients who express a preference for telephone consultations may reduce patient burden by eliminating the need for potentially unnecessary travel to hospital should intervention be successful, particularly for patients who lack independence or those who are working.

At the time of writing, we are currently facing unprecedented challenges in relation to the COVID-19 pandemic with many services traditionally delivered face-to-face health services transitioning to virtual/ telephone consultations in order to minimise hospital visits and physical patient-clinician contact and thus virus transmission [13]. This clinical audit was initially conducted from January 2015 to May in 2015. In light of the current challenges being faced in clinical practice and the significant adjustments being made to facilitate ongoing service delivery, the authors felt publishing our methods and findings from this clinical audit at this time would be beneficial. The implementation of virtual consultations within NHS Trusts in the wake of the pandemic has been strongly advocated recently [19]. It is likely that some increase in reliance on telehealth will persist and identifying those patients who do not require face-to-face appointments will prevent unnecessary hospital visits, while offering clinicians the opportunity to provide the face-to-face appointments to those patients whose symptoms genuinely require face-to-face review.

Telephone follow-up services have been shown to have no detrimental impact on patient satisfaction [20–22]. However, given the structured approach of the questions used within this telephone follow-up system in contrast to more traditional semi-structured consultations adopted in such studies, the impact of this on patient satisfaction is unclear. Should this system be implemented more widely, a subsequent audit should focus on evaluating patient satisfaction in order to evaluate this. Patient involvement in the development of new services has been advocated for in the literature and clinical guidelines in the UK [23]. Services aiming to adopt a similar approach to the one outlined in this clinical audit should consider this in order to maximise patient satisfaction and to identify potential barriers to successful delivery. If implemented in practice, a subsequent evaluation of changes in appointment waiting times, real-time calculation of cost savings and the number of patients requiring subsequent face-to-face review would also be prudent.

## Conclusions

The use of a telephone-based follow-up consultation service within a Rheumatology Podiatry Department may offer a potentially time and cost-efficient alternative to traditional face-to-face consultation appointments for people with RMDs, although the latter will be heavily influenced by local commissioning contracts. Integrating these alongside a traditional approach to service delivery could improve patient throughput by increasing the availability of follow up face-to-face consultation appointments. Adopting an approach such as the one presented within this clinical audit, underpins the need to minimise non-essential face-to-face consultations during the current COVID-19 pandemic. Offering telephone follow-up consultations is likely to be most efficient if they are arranged prospectively to reduce potential losses to follow-up and to improve the efficiency of making successful contact with patients. Research is required to evaluate patient satisfaction of telephone-based follow-up consultations in this clinical setting.

## Supplementary Information

**Additional file 1.**

## Data Availability

The datasets used and/or analysed during the current study are available from the corresponding author on reasonable request.

## Abbreviations

RMD: Rheumatic and musculoskeletal diseases
ARMA: Arthritis and Musculoskeletal Alliance
PRCA: Podiatry Rheumatic Care Association
NICE: National Institute for Clinical Excellence
NWCEG: North West Clinical Effectiveness Group
LTHT: Leeds Teaching Hospitals NHS trust
UK: United Kingdom
GRC: Global Rating of Change
RCT: Randomised Controlled Trial
MCID: Minimal Clinically Important Difference
