# Supplementary material for: Implementation of podiatry telephone appointments for people with rheumatic and musculoskeletal diseases
Source: J Foot Ankle Res. 2021 Jan 7;14:4. doi: 10.1186/s13047-020-00441-9 (PMC7790049; doi:10.1186/s13047-020-00441-9)
Supplement: Supplementary file 1 — Additional file 1. [file 13047_2020_441_MOESM1_ESM.docx]

Appendix 1: Telephone Consultation Questions

1. How have your foot symptoms felt since being provided with your foot health intervention? Better, Same or Worse?’

2. ‘How would you rate the change in your symptoms on the following scale?’

-7: A very great deal worse
-6: A great deal worse
-5: A good deal worse
-4: Moderately worse
-3: Somewhat worse
-2: A little worse
-1: Almost the same, hardly any worse at all
0: No change
1: Almost the same, hardly any better at all
2: A little better
3: Somewhat better
4: Moderately better
5: A good deal better
6: A great deal better
7: A very great deal better

3. ‘Do you feel you require a further appointment in the rheumatology foot health clinic?’

4. ‘Would prefer to receive a follow-up telephone consultations or face-to-face clinic appointment?’
